# Anatomy in present times: A satire

**DOI:** 10.12669/pjms.38.4.5799

**Published:** 2022

**Authors:** Dr. Zilli Huma

The one constant in this world is change and one of the biggest examples of this is the process of teaching human anatomy to our latest generation of doctors. Do we call them the zoom generation now? Bear with me as we explore and dissect out the changes and challenges of learning this basic of all medical subjects over the past century and the challenges faced.

I present the analogy of “The comedy of errors”. This play by William Shakespeare embodies the trials and tribulations of members of a family separated and then united, i.e., Anatomy from the start of modern medicine to present times and the tale though tragic, becomes comic in its absurdity.

The case is presented of the mistaken identity when bodies were stolen for dissection in anatomy schools: a news article (1818) tells us about body snatchers disturbed in the Chebacco cemetery, Massachusetts where the general assumption was that there was no hope of recovery as the bodies had probably “passed under the dissecting knife of the anatomist”.^1^ People made a living out of grave digging as illustrated by an 1887 obituary in the Washington Post of the death of one William Jansen, “a king among ghouls”.^2^ Benjamin Brodie (Personal surgeon to the King of England) also placed an emphasis on dissection saying, “each student should have to dissect five bodies over the course of his training”.^3^ The public labelled these doctors and anatomy schools as grave robbers and even murderers. So, anatomists were stuck in the middle of a controversy of dissection being essential for the modern doctor and the paucity of lawful ways and means to procure these. This was a catalyst for legislation as a way out of this dilemma, a compromise to appease the public and ensure enough bodies to satisfy the medical schools. Thus, the 1832 Anatomy Act (UK) stated that poor unclaimed bodies can be used by licensed anatomists with the subsequent 1854 Bone bill (USA) that stated: unclaimed bodies may be used by anatomists for dissection. This helped create a respectable identity for the anatomist from being gravely mistaken to dissection as usual within the law. This helped pave the way to a regulated body donation program in most countries. Though sadly lacking in our country.

The next game changer in learning medicine came as a case of near seduction where different associations and accrediting bodies came together to develop a structure for licensing of medical practitioners. Specialty societies collaborated with the American Congress of Physicians and Surgeons to work on the council of medical education wherein 1907, they divided medical schools into classes A, B and C depending on their ratings. On the surface, this appeared as a progressive step, but the long-term outcomes brought about discrimination and power play by private wealthier schools. A hundred years, later PM&DC in Pakistan repeated the same whereby giving a lot of sway to the clinical side, i.e., College of Physicians and Surgeons Pakistan in collaboration with clinical specialty societies and Pakistan Association of Private Medical and Dental Colleges (PAMI). The Pakistan medical council (PMC) then proved even more devastating for basic medical sciences, removing MPhil and PhD from PMC registration. The absurdity is highlighted by the fact that a master’s in public health has now been designated as a clinical specialty and can be registered in PMC. A doctor with post graduate qualifications of MPhil and PhD in anatomy cannot register in PMC anymore and are deemed academic degrees to be registered by Higher Education Commission (HEC). This has created a general sense of unease in basic medical teachers and scientists that has made them file multiple petitions against this decision. The context and differences in our situation to the western world needs to be understood. The idea of having full time dedicated faculty for medical schools was started at John Hopkins in 1893, with emphasis on research where universities were mandated to provide full-time faculty positions in anatomy, physiology, pathology, and pharmacology. The physicians on the other hand were given a choice of gold or glory. The argument was that clinical departments should be headed by physicians that are “properly paid and of whom more may be demanded than of those who regard their clinical services merely as a means of rapidly acquiring a large private clientele. “(AMA Journal, 1900). Thus, an analytical approach rather than experimental would have been a more logical approach in our country before removing registration of basic science post graduate qualification from PMC.

The case for infidelity is more ambiguous, case in point the Flexner report.^4^ This report funded by the Carnegie foundation brought about the great purge of American medical schools.^5^ Thus money played another crucial role in shaping medical education with huge donations given out to schools under the umbrella of reform for the idea to get approved. A conflict of interest in many ways. Though it had many positive aspects where there was a conscious effort to streamline and structure the training of medical students, engaging medical faculty in research and giving schools control of clinical instruction in hospitals all to strengthen state regulation of medical licensure. Preventive medicine and population health were not considered a responsibility of physicians, bifurcating “health” into two separate fields: scientific medicine and public health. Narrowing the scope and training of young doctors.

We have learnt that with precise scientific knowledge there is an element of care and service that should be part of a doctors training.^6^ So steps were taken towards global standards for all medical schools to increase mobility of doctors all over the world and ensuring acceptable health safety standards for the public. To ensure this PMC standards were then developed according to the World Federation of Medical Education (WFME). One should keep in mind that these are guidelines and should be taken in context of our cultural and situational differences. The mandate for our population may slightly differ in terms of disease understanding and we should be wary of importing foreign concepts and implementing them into our system absolutely. Rather than a purge a more feasible and appropriate approach would be to integrate and tailor these concepts to our situations. For example, the latest gadget in anatomy learning are virtual simulated dissecting tables which may not be affordable to many colleges, so do we shut them down?^7^

The final nail in the coffin of anatomy learning and teaching is a case of demonic possession. How else to describe “anyone can teach medicine”? Agenda point 9 placed before the PM&DC, the council did not agree to the proposal to confine the faculty of medical and dental colleges to holders of MBBS or BDS only. The council agreed that colleges/universities can employ outstanding teachers of appropriate basic science subjects to teach in medical and dental colleges and an MBBS or BDS qualification for these would not be mandatory. The ambiguity lies in words “outstanding” and “appropriate”. The question to be asked is how is it possible, for a non-medic with a PhD in Anatomy to be able to teach students human anatomy at the application level. A PhD as everyone understands is detailed knowledge in a very niche area of study. Thus, the position of all basic science teachers has become ambiguous see sawing between PMC and HEC.

In conclusion the curtain closes on this comedy of errors of gravely mistaken identities, near seduction translated to near sightedness, infidelity to injustice and demonic possession as dementia possibly. Result, is confusion and chaos. But keeping in mind the Comedy of errors had a happy ending, and for this communication and understanding are the key differentials. We all have the same goal, that our future generation of practitioners be knowledgeable and safe professionals. Therefore, it is high time to take a step back and analyse the impact of our actions enacted today on the health ecosystem of Pakistan 50 years from now..
